# Cardiovascular risk escalation with caloric excess: a prospective demonstration of the mechanics in healthy adults

**DOI:** 10.1186/1475-2840-12-23

**Published:** 2013-01-24

**Authors:** Alok K Gupta, William D Johnson, Darcy Johannsen, Eric Ravussin

**Affiliations:** 1 Clinical Research, Pennington Biomedical Research Center, Louisiana State University System, 70808, 6400 Perkins Rd,, Baton Rouge, LA, USA; 2 Basic Research, Pennington Biomedical Research Center, Louisiana State University System, 70808, Baton Rouge, LA, USA; 3 Population Research, Pennington Biomedical Research Center, Louisiana State University System, 70808, Baton Rouge, LA, USA

**Keywords:** Fasting plasma glucose, Healthy adults, Visceral adipose tissue, Ectopic adipose tissue, Fat cell size, Insulin resistance, Systemic inflammation, Dyslipidemia, Predisease conditions, Circadian blood pressure variability, Relative hyperemia index, Endothelial dysfunction

## Abstract

**Context:**

The link between weight gain and cardiovascular risk characterized with circadian blood pressure variability [CBPV] and endothelial function [EF] is unexplored.

**Objective:**

To prospectively demonstrate weight gain in healthy adults, increases body fat [BF], enlarges waist circumference [WC], expands visceral adipose tissue [VAT], exacerbates systemic inflammation [sIF], worsens insulin resistance [IR] and enhances functional cardiovascular disease [CVD] risk.

**Design, setting and participants:**

Healthy men [n=11] and women [n=3] provided initial and eight-week post-caloric excess anthropometric and fasting laboratory measures. Functional CVD risk assessments: CBPV and resting EF were also obtained with 7-day automatic ambulatory BP monitoring and increased test finger peripheral arterial tone [PAT] relative to control [reported as relative hyperemia index (RHI)], respectively.

**Intervention:**

After determining individualized mean energy requirements for weight maintenance over 7-days, each participant received a personalized over feeding prescription (1.4 times; 41% carbohydrate, 44% fat, and 15% protein) for 8-weeks.

**Results:**

mean (SEM). Participants increased body weight [BW; +7.4(0.1) kg]*, body mass index [BMI; +2.5(0.2) kg/m^2^]*, BF [+2.0(0.01)%]*, WC [+8.2(1.0) cm]*, and VAT [+0.2(0.03) L]* and intrahepatic lipid [IHL + 0.0004(0.002) L] :*all p < 0.01. Increased subcutaneous adipose cell size [+0.3(0.01) ρL; p = 0.02] accompanied significant sIF [hs-CRP + 0.4(0.09) mg/dL; p = 0.04; leptin 6.63 ng/ml; p = 0.0008] and IR [fasting plasma glucose; [FPG] +7.0(0.6) mg/dL;p = 0.01, fasting insulin; [FI] +5.7(1.4) uIU/ml; p = 0.001, HOMA-IR +1.6(0.5); p = 0.02]. Abn CBPV {systolic [+5.4(0.8); p = 0.002, diastolic [+1.7(0.1); p = 0.07 and pulse pressure [PP] [+3.5(0.4); p = 0.003 mm Hg} or elevated heart rate [HR] [+4.9(0.5) bpm; p = 0.003] ensued. Resting RHI declined by 0.47(0.004) from initial 2.24(0.09) to 1.77(0.1); p = 0.001, indicating endothelial dysfunction [ED].

**Conclusions:**

Controlled caloric excess in healthy human adults over only 8-weeks significantly increased BF, VAT, sIF [hs-CRP], IR [FPG, FI, HOMA-IR] and functional CVD risk [measured as abnormal circadian blood pressure variability and impaired resting endothelial function].

## 

Excess adipose tissue, which is a hallmark for an overweight or obese status [1,2], in ectopic locations [like the visceral compartment, liver, muscle] is dysfunctional. The secreted adipokines [including cytokines and chemokines] which mediate auto, para and endocrine actions, alter the dynamic homeostatic milieu to favour systemic inflammation, and often manifest dysglycemia, dyslipidemia, and/or a loss of blood pressure control [3-6]. These early changes clinically seen as prediabetes, premetabolic syndrome and/or prehypertension, as many as 10-15 years in advance of the chronic changes subsequently leading to a diagnosis of noncommunicable diseases like diabetes mellitus and hypertension [4,7]. The early increase in pro-inflammatory and pro-coagulant factors, reactive oxygen species, dysglycemia, dyslipidemia, and loss of blood pressure [BP] control, are functionally detectable as abnormal circadian blood pressure variability [abnCBPV] and resting endothelial dysfunction [rED] [8,9].

Asymptomatic overweight adults with prediabetes, [compared to matched overweight adults with normal glucose], manifest abnormal circadian blood pressure variability [10]. Among asymptomatic obese adults only those with prediabetes and highly exacerbated systemic inflammation, [compared to matched obese with normal fasting glucose and marginally elevated systemic inflammation], display both abnormal circadian blood pressure variability and resting endothelial dysfunction [8]. Prediabetes and prehypertension in otherwise healthy adults singly, or together [co-existing prediabetes and prehypertension] place the individual on a pathway with potential for accelerated cardiovascular adverse events, including sudden death [11-14].

These results led us to formulate our overall hypothesis that caloric excess, even in the short term, results in ectopic adipose tissue deposition. The adipose tissue cells enlarge, alter their adipokine secretion menu tipping the pro-inflammatory and anti-inflammatory homeostatic balance in favour of inflammation [leading to altered glycemia and lipidemia] and potentiating the elements of the renin-angiotensin-aldosterone system [resulting in loss of BP control]. This latent, but high cardiovascular risk is clinically manifest as prediabetes and/or prehypertension and functionally as abnormal circadian blood pressure variability and resting endothelial dysfunction.

With a well designed experiment, the early relationship between caloric over-loading [modelled after the usual American diet], an increased systemic inflammation, fasting plasma glucose and loss of blood pressure control was elucidated in genetically permissible rats. We were able to show that both systemic inflammation and glycemia influence circadian blood pressure variability. We were further able to demonstrate that dysglycemia induces abnormal circadian blood pressure variability [15].

This prospective study with baseline and eight-week- post controlled caloric excess measurements tests our hypothesis that significantly expanded visceral adipose tissue [increased body mass index and waist circumference], increases adipose cell size [signifying adipose tissue dysfunction], potentiates systemic inflammation [increased C reactive protein, leptin] and worsens insulin resistance [elevated fasting plasma glucose, fasting insulin, and Homeostasis Model of Assessment-Insulin Resistance (HOMA-IR)] in healthy adults. These changes are also accompanied by persistent abnormal circadian blood pressure variability and endothelial dysfunction, both of which are functional correlates indicating enhanced cardiovascular risk.

### Methods

#### Study design and population

The EAT [Fat Cell Size, Overfeeding and Ectopic Fat] study was approved by the Pennington Biomedical Research Center Institutional Review Board and all volunteers provided written informed consent at the first screening visit. Participants ages 20–40 years with BMI 22.0–32.0 kg/m^2^ and no history of chronic disease [high blood pressure, diabetes, severe obesity, heart, kidney, liver or gastrointestinal disease] were enrolled. These subjects were willing to participate in an 8-week study that resulted in a 5 – 10% weight gain, consume all meals at the Pennington Biomedical Research Center and not change their usual level of physical activity. This manuscript includes data for fourteen [of a total of thirty five] subjects who consented for additional 7-day ambulatory blood pressure monitoring and resting endothelial function testing.

#### Study methods

The study methods are described in detail previous publications [8,10] and in the main paper which includes all thirty five subjects from this study. A brief description of the methods is as below.

##### Initial screening

Included anthropometric measures [height, weight: for calculation of body mass index], cardiometabolic testing [complete blood count, chemistry-15 panel included lipid profile, hepatic and renal function measures], serum insulin and preparation of the subjects for overfeeding. An archived plasma sample was used at the end of the study to measure markers of systemic pro-inflammatory [high sensitivity C reactive protein], leptin and anti-inflammatory activity [adiponectin].

###### Over feeding

A study dietitian discussed types and volume of food that were to be provided during the 8-week overfeeding phase, along with the requirement for consuming all meals on-site, under direct supervision. A typical meal was presented to give the potential subject a visual impression of the type and volume of food to be consumed.

##### Second screening

Comprised of the study physician performing due medical diligence. A detailed medical history followed by a complete physical examination, along with a review of the cardiometabolic testing was performed by the medical investigator. This screening visit also included repeat fasting blood draw for cardiometabolic testing, urinalysis and a pregnancy test [for females].

##### Run-in testing

After successful completion of both screening visits, subjects were enrolled in the study and were scheduled for a 2-week run-in period. The purpose of the run-in phase was to determine baseline energy requirements and perform baseline testing. Included [week 1 of run in] were a weight-specific dose of doubly labeled water to enable measurement of energy expenditure and activity monitor, along with a record of all food [solid and liquid] intake. Subjects were advised to eat normally, and reported to Pennington Biomedical Research Center [PBRC] every morning for a fasted metabolic weight [after voiding and in a hospital gown].

Beginning on day 8 [week 2 of run-in], subjects were provided all meals according to their estimated calorie requirements. Breakfast and dinner were consumed under supervision in the inpatient unit, while lunch was packed to go. The diet consisted of 41% carbohydrate, 44% fat, and 15% protein. Participants were instructed to eat all of the provided food and to record any additional items consumed. Metabolic weight was obtained every day prior to breakfast.

##### Baseline and post overfeeding testing

To proceed to baseline or post overfeeding testing, participants had to be in energy balance [± 0.5 kg] for 3 consecutive days. For the baseline and post-overfeeding testing, participants remained on the PBRC inpatient unit for 3 consecutive days, during which they completed the following tests.

###### Metabolic chamber

Sedentary energy expenditure was measured over 23 hours in a whole-room calorimeter.

###### Subcutaneous adipose tissue biopsy

After exiting the chamber, under fasting conditions, participants underwent subcutaneous fat biopsy. Approximately 300 mg of subcutaneous adipose tissue was collected by Bergstrom needle for measurement of fat cell size, followed by an additional 1,000 mg of tissue collected by liposuction for numerous RNA, DNA, histology, and protein measurements.

###### Magnetic resonance imaging [MRI]

Total abdominal adipose tissue including visceral [VAT] and subcutaneous [SAT] was measured using a 3.0 T scanner [General Electric, Excite HD System, Milwaukee, WI].

###### Dual energy X-ray absorptiometry [DXA]

Whole-body percent fat was measured by DXA [Hologics QDR 4500A; Hologics, Bedford, MA]. Fat mass [FM] and fat-free mass [FFM] were calculated from the percent body fat and measured metabolic body weight.

###### QuickScan

A quick direct measurement of the amount of fat, muscle, and water in the body was obtained using a magnet.

###### ^1^H Magnetic Resonance Spectroscopy [MRS] for ectopic lipid

Lipid content of the soleus and anterior tibialis muscles [intramyocellular lipid, IMCL] and liver [intrahepatic lipid, IHL] were measured by ^1^H MRS using the Point Resolved Spectroscopy [PRESS] box technique. Liver fat was measured with a commercially built ^1^H body coil.

###### 7-day ambulatory blood pressure monitoring and resting endothelial function testing

This testing was performed during the week 2 of the run-in period.

###### Blood pressure and heart rate measurement

An ambulatory blood pressure monitoring device [Mortara® Ambulo 2400] for ambulatory use was attached to a BP cuff to obtain BP and HR readings at 30-min intervals during the day [6:30 AM to 9:30 PM] and 60-min intervals at night [10 PM to 6 AM] while the participants went about their activities [8,10]. Data were downloaded into the database at the end of the 7-day recording span for a chronobiological analysis [8,10].

###### Endothelial function testing

Assessment of resting endothelial function was done with the participant in a fasting state, after having avoided stimulants [caffeine, tobacco, alcohol, exercise] for 12 hours, at the same fixed clock hour [range 8-10 AM], using the EndoPAT 2000 device manufactured by ITAMAR Medical®. This assessment technique has been previously validated [16], has been used in numerous [>250] peer reviewed publications [9,17,18] and has been in routine use in our clinical core. Briefly: subjects coming in from home, after an overnight fast and having avoided stimulants for 12-hours, were placed in a supine position for 20 minutes in a quiet room before the test. A patented single use finger sleeve was then placed on the index finger of each hand to continuously measure peripheral arterial tone. A blood pressure cuff applied to the upper arm of the non-dominant arm (test arm) was then used to occlude the brachial artery for 5 minutes. This was followed by a rapid release. The dominant arm without any manipulation served as the control. The built in, validated software integrated the data gathered from the finger sleeves of the control (undisturbed) and the test arms (during the baseline, occlusion and release phases), thus providing the relative hyperemia index (RHI) for the test arm. This flow mediated dilatation induced change in the test arm, relative to the control arm, served as the measure for endothelial function and was expressed as relative hyperemia index [RHI].

##### Overfeeding

Participants were allowed one day of recovery from baseline testing prior to beginning overfeeding. Prior to overfeeding, all participants completed a 2-week measure of free-living energy expenditure by doubly labeled water (DLW). During the second week of DLW, participants were fed to energy balance with a diet consisting of 60% carbohydrate, 25% fat, and 15% protein. Energy requirements to maintain usual body weight were determined from the run-in period and calculated as the 7-day average of the number of kcals required for energy balance and the 14-day number of kcals expended as measured by doubly labeled water [DLW]. This number was then multiplied by 1.4 to obtain the subject’s overfeeding prescription, and this individual prescription was maintained throughout all 56 days [i.e. there was no ramp-up period or re-adjustment for body weight changes]. All meals were prepared in the metabolic kitchen at PRBC using a validated five-day rotating menu. The diet consisted of 41% carbohydrate, 44% fat, and 15% protein for all subjects. Participants were free-living during the overfeeding period but were required to report to PBRC three times per day, seven days per week to consume their meals, which were directly supervised by nursing staff to ensure that all foods were eaten. During the final days of the study prior to metabolic testing, subjects were fed to energy balance with the same diet composition (60% carbohydrate, 25% fat, and 15% protein) provided at baseline.

###### Post overfeeding testing

Subjects had to be weight stable at their new [highest] body weight for 3 days [±0.5 kg] prior to beginning post-overfeeding testing [average 3 to 5 days]. On day 56, the number of kcals needed to maintain energy balance at each subject’s new body weight was calculated according to the equation used during the run-in period. For 3 days following overfeeding [days 57 – 59] subjects reported to PBRC as usual but were provided with their new energy balance diet, which consisted of 60% carbohydrate, 25% fat, and 15% protein. Once stable, participants were admitted to the inpatient unit for 3 days of post-testing, which consisted of repeating all baseline tests. Subjects were discharged from the unit with appointments to return for follow-up testing at 1, 3 and 6 months later [not included in the present analysis].

###### Adipose tissue measurement: fat cell size

Adipocyte cell [AC] size was measured using methods adapted from Hirsch and Gallian [19].

###### Statistical analyses

All data analyses were conducted using SAS version 9.3 [SAS Institute, Inc., Cary NC]. Categorical data were summarized as counts and percentages whereas measurements assessed on continuous scales were summarized as means and standard errors. Statistical significance [p ≤ 0.05] of changes observed after eight weeks of excess caloric intake was evaluated using paired t-tests. Correlation coefficients were calculated to assess pair-wise associations between changes in response to excess caloric consumption.

### Results

#### Demographic, anthropometric, body composition and metabolic characteristics of the participants

All results are expressed as mean(SEM). These otherwise healthy 14 participants, 3 women and 11 men, 6 African American/8 Caucasians on average were 28.4(1.1) years young, weighed 79.4(3.4) kg and had BMI of 25.6(0.07) kg/m^2^. Their mean weight by QuickScan was 79.1(3.3) kg, with 56.7(3.3) kg of lean mass and 15.3(1.8) kg of fat mass. Their mean WC was 82.5(2.2) cm; women with mean 75.1(3.3) cm and men with mean 84.6(2.3) cm, well within the desirable range of <88 and <102 cm, respectively. They exhibited mean 22.2(2.2)% body fat by a DXA assessment with men having a lower mean at 18.7(1.4)% and women, as is customary, having a considerably higher 34.91(1.03)% fat. A subcutaneous AT biopsy revealed mean AC size of 0.596(0.09) ρL. Men displayed smaller [0.58(0.1) ρL] adipose cells, compared to women [0.64(0.01) ρL].

A complete blood count, fasting serum chemistry including hepatic functions [Alkaline phosphatase 52(4.6) IU/L and Alanine transaminase (20(1.5) IU/L] and kidney functions were all normal. They all had normal FPG [90.6(1.7) mg/dL], FI [4.7(1.0) μU/ml] and insulin resistance [HOMA-IR 1.06(0.22)]. Their fasting lipid profile [mean Total-C 177(7.6), LDL-C 102(7.1), HDL-C 60(3.4) and TG 75.1(9.7) mg/dL] indicated metabolic normality.

#### Clinical characteristics of the participants

All subjects were healthy as indicated by a complete medical history [including past medical, surgical, social and family history] and a comprehensive medical examination. This was performed by the study physician (medical investigator). None of the participants were on any prescription medications or had any chronic medical conditions requiring chronic intake of prescription medications. One, two and three participants had a family history of coronary artery disease, diabetes mellitus and hypertension, respectively. These subjects at their clinic visit had a normal temperature [36.6(0.3)°C], resting BP [116.8(8.5)/75.2(6.6) mm Hg], PP [41.6(3.8) mm Hg] and HR [55.7(8.7) beats per minute]. The mean BP, PP and HR by ABPM over 7-days were: SBP/DBP 110.7(2.5)/67.8(1.6) mm Hg, PP 42.9(0.9) mm Hg and HR 70.3(2.3) beats per minute. The resting EF, measured in the morning [between 8-10 AM] in a fasting state after having avoided stimulants [caffeine, tobacco, alcohol] for over 12 hours was RHI 2.24(0.09). Table 1 details demographic, anthropometric, body composition, laboratory and clinical characteristics for the participants at baseline.

**Table 1:** Demographic, anthropometric, body composition, laboratory and clinical characteristics of participants at baseline

			
AGE (years)	28.3(1.12)	hs CRP (mg/L)	1.2(0.26)
GENDER (M/F)	11M/3F	Leptin (ng/ml)	13.0(16.1)
RACE (C/AA)	8C/6AA	Adiponectin (μg/ml)	4.6(2.4)
BMI (kg/m^2^)	25.6(0.65)	FPG (mg/dL)	90.6(1.72)
WC (cm)	82.5(2.15)	FI (μU/ml)	4.7(0.99)
FAT MASS (kg)	15.3(1.81)	HOMA-IR	1.1(0.22)
LEAN MASS (kg)	56.7(3.27)	SBP (mm Hg)	110.6(2.5)
VAT (L)	0.5(0.18)	DBP (mm Hg)	67.8(1.5)
IHL (L)	0.005(0.001)	PP (mm Hg)	42.9(2.6)
ALT (IU/L)	20.1(1.53)	HR (bpm)	70.3(2.3)
SAT CELL SIZE (ρL)	0.59(0.09)	EF (RHI)	2.24(0.09)

MEAN(SEM), *BMI* Body Mass Index, *WC* Waist Circumference, *VAT* Visceral Adipose Tissue, *IHL* Intrahepatic lipid, *ALT* Alanine transaminase, *SAT* Subcutaneous Adipose Tissue, *FPG* Fasting Plasma Glucose, *FI* Fasting insulin, *HOMA-IR* Homeostasis Model of Assessment-Insulin Resistance, *SBP* Sytolic Blood Pressure, *DBP* Diastolic Blood Pressure, *PP* Pulse Pressure, *HR* Heart Rate, *EF* Endothelial Function.

#### Cardiovascular disease risk

Traditional cardiovascular risk factors, both non-modifiable [age, gender, heredity] and modifiable [obesity, cigarette smoking, high blood pressure, diabetes, elevated serum total cholesterol [Total-C], low high-density lipoprotein cholesterol [HDL-C], high triglycerides [TG], glucose intolerance, low to high-density lipoprotein cholesterol ratio] were assessed. The 10 year CVD risk was calculated for each participant using the National Cholesterol Education Program, Adult Treatment Panel III criteria. This was found to be <1% for each participant.

#### Post caloric excess anthropometric, body composition measures

These healthy subjects during 8-weeks of over feeding on average gained weight [+7.4(0.1) kg; p = 0.001], increased body fat [+2(0.01)%; p = 0.001], fat mass [+4.5(0.2) kg; p = 0.001], body mass index [+2.5(0.03) kg/m^2^; p = 0.001] and waist circumference [+8.2(0.1) cm; p = 0.001]. The increase in waist circumference +8.3(4.6) cm in women and +8.2(2.3) cm in men, however, did not place the mean for either group above the desirable range for the waist circumference (>88 and 102 cm for women and men, respectively]. On average visceral adipose tissue increased [+0.25(0.03) L; p = 0.01], intramyocellular lipid measurements decreased [-0.00061(-0.00023) L], but intra hepatic lipid increased [+0.0042(0.0025) L]. Figure 1 details the change in body composition

**Figure 1:**
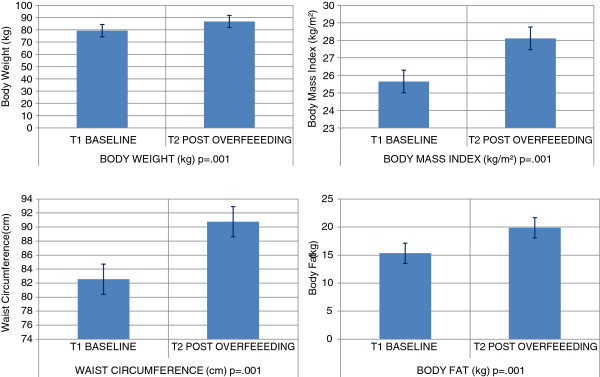
Change in body composition.

#### Post caloric excess subcutaneous fat cell size

On average subcutaneous adipose cell size increased significantly from 0.596(0.91) to 0.919(0.1) ρL; p = 0.02.

#### Post caloric excess elevation in systemic inflammation

Systemic inflammation assessed by high sensitivity C reactive protein [hs CRP], although still within the normal range, increased significantly on average from 1.24(0.25) to 1.6(0.45) mg/L. Another marker for systemic inflammation, serum leptin increased significantly from 13.03 to 19.66 ng/ml; p = 0.0008. Non-specific markers for inflammation like the total white blood cell count and serum uric acid levels, although marginally higher, did not change significantly.

#### Post caloric excess glycemia, insulin resistance, adiponectin, lipid profile and hepatic functions

Fasting glucose significantly increased by an average of 7.0 mg/dL [range +31 mg to -6 mg/dL; p = 0.02], analogous increases were observed for fasting insulin at 5.7 μU/ml [range +22.9 to +0.6 μU/ml; p = 0.04] and insulin resistance [HOMA-IR +1.6; range +8.0 to +0.2; p = 0.04]. Although significant changes were not observed in the complete blood count, fasting serum chemistry and adiponectin [anti-inflammatory adipokine], significant increases in the fasting lipid profile [Total-C +18.9(15.7), LDL-C +13.5(12.5), HDL-C +5.3(6.7); p < 0.01] with the exception of TG [+0.7(18.5) mg/dL] were noted. The biomarker for hepatic function increased significantly serum Alanine transaminase (ALT) [32.9(17.4) IU/L; p = 0.01]. Figures 2 and 3 depict the changes in body adipose tissue distribution and change in systemic pro inflammation and glycemia, respectively.

**Figure 2:**
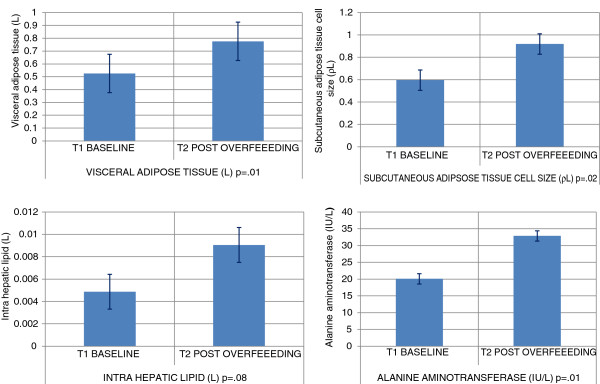
Change in body adipose tissue distribution.

**Figure 3:**
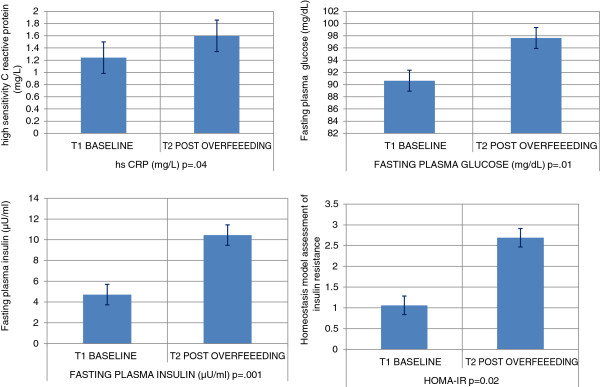
Change in systemic pro inflammation and glycemia.

#### Post caloric excess clinical and circadian blood pressure changes

The clinic visit temperature and means of resting blood pressure did not change significantly [36.6(0.3)°C, SBP/DBP 118.8(10.3)/74.4(7.4) mm Hg], but the mean resting heart rate significantly increased from 55.7(8.6) to 66.3(12.2) beats per minute, p = 0.02. The mean systolic and diastolic blood pressure, pulse pressure and heart rate measured by ambulatory blood pressure monitoring over 7-days, however, all exhibited significant increases. The overall group mean systolic blood pressure, diastolic blood pressure and pulse pressure increased +5.4(0.8); p = 0.002, +1.9(0.1); p = 0.07, +3.5(0.4); p = 0.003 mm Hg, respectively. There was also a significant increase in heart rate +4.9(0.5); p = 0.003 beats per minute. There were significant increases in individual post caloric excess means for systolic blood pressure (9 out of 14), diastolic blood pressure (5 out of 14), pulse pressure (7 out of 14) and heart rate (7 out of 14). Every participant (with the exception of two) had one, or more than one (post caloric excess over baseline) significant change in the above circadian blood pressure variability measures.

#### Post caloric excess endothelial function

The resting endothelial function measurement was performed in 12 of the 14 volunteers. The resting endothelial function declined with relative hyperemia index decreasing from 2.24(0.35) to 1.77(0.36); p = 0.004 in 10 of the 12 subjects. The remaining two subjects could not have their baseline assessment due to equipment malfunction. Figure 4 illustrates the change in functional cardiovascular risk.

**Figure 4:**
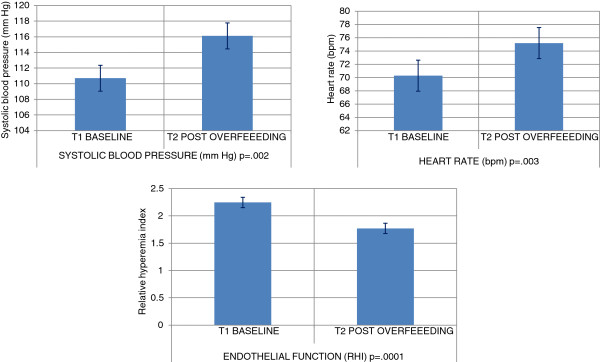
Change in functional cardiovascular risk.

#### Correlations

Body composition measures exhibited widely known and established correlations. Body mass index was strongly correlated with body weight [r = 0.93], while waist circumference was correlated with both body weight [r = 0.50] and body mass index [r = 0.61].

Circadian blood pressure variability measures were correlated with each other. Mean diastolic blood pressure was correlated with mean systolic blood pressure [r = 0.68]. Mean double amplitude of variability of diastolic blood pressure was correlated with the analogous variability of systolic blood pressure [r = 0.80] and pulse pressure was correlated with 7-day mean heart rate [r = 0.51].

Body weight was correlated with 7-day mean heart rate [r = 0.44], while body mass index was correlated with 7-day mean heart rate [r = 0.42] and mean PP [r = 0.41]. Waist circumference was correlated negatively with standard deviation of heart rate [-0.55].

Systemic pro inflammation was highly correlated with 7-day mean systolic blood pressure [r = 0.61], pulse pressure [r = 0.58], body weight [r = 0.82] and body mass index [r = 0.84].

Fasting glucose was correlated with standard deviation of heart rate [r = 0.46] and subcutaneous adipose tissue fat cell size [r = 0.46].

Total-C [r = 0.62], LDL-C [r = 0.45] and HDL-C [r = 0.55] were correlated with standard deviation of heart rate.

Triglycerides were correlated with visceral adipose tissue mass [r = 0.71]. Alanine transaminase was correlated with 7-day mean heart rate [r = 0.74], standard deviation of heart rate [r = 0.54] and visceral adipose tissue mass [r = 0.48].

Endothelial function was negatively correlated with mean fasting plasma glucose [r = -0.40], total-C [r = -0.55], LDL-C [r = -0.57] and triglycerides [r = -0.51].

### Discussion

All fourteen participants after eight weeks of caloric excess exhibited increase in weight ranging from 2.7 to 11.1 kg, with a mean gain of +7.4(2.5) kg. The mean gain in fat which was 4.5 kg (range 3.0-6.3 kg), translated to an increase of 2% in body fat (range -0.15-4.9%) and the lean mass by 2.82 kg (range 0.8-4.4 kg). These changes along with the increase in body mass index, waist circumference were all significant. The American Heart Association uses body mass index for assessment of total body adiposity and waist circumference as a marker for abdominal body fat [20]. In a biracial sample of men and women common anthropometric measures (including body weight, waist circumference and body mass index) were moderately or highly correlated with total body fat, abdominal fat and cardiovascular disease risk factors [21]. These indices have also been used in the discovery of out of the normal range blood pressure in adults, and both out of normal range blood pressure and lipids in adolescents [22,23]. Waist circumference is a surrogate measure for visceral adipose tissue which has clinical utility as an independent predictor of all-cause mortality [24]. But despite these strong correlations, gender and race influences these interpretations [25,26]. Excess adipose tissue in the visceral compartment has been associated with increased CVD risk in large prospective studies: the Framingham and the Jackson Heart studies [27,28]. In the present study with the overall body weight gain we were able to demonstrate a significant increase in body fat [+2%] and visceral adipose tissue volume [+0.2(0.03) L]. The increase in visceral adipose tissue far exceeded the increase in subcutaneous tissue (103% vs 30%), indicating a preferential deposition in the visceral compartment.

Caloric excess, which results in body weight gain and expansion of the visceral adipose tissue compartment, can also initiate an obligatory adipose tissue remodeling [29]. The changes beginning with the initial expansion to maturation of adipocytes, followed by the infiltration of macrophages, onset of hypoxia, inflammation and development of insulin resistance have been reviewed in exquisite detail [30]. We elucidated a significantly increased mean subcutaneous adipose cell size [+0.32 ρL] is these participants subsequent to 8-weeks of caloric excess. While this is not a direct measure of the visceral adipose cell size, it is an indication of a mature subcutaneous adipose cell, at capacity, with no additional room for storage. Indeed the magnitude of increase in visceral adipose tissue (103%) compared to the subcutaneous adipose tissue (30%), attests to preferential deposition in the visceral compartment. Since a mature large adipose tissue cell is dysfunctional, and there is evidence of dysfunction attributable to the visceral adipose tissue, the increase in visceral adipose cell size could be inferred.

The adipocyte hyperplasia and hypertrophy which contribute to adipose tissue expansion can also result in visceral adipose tissue dysfunction [30]. The exacerbated systemic inflammation in obesity often results in increased insulin resistance [12,31]. Non-specific makers for systemic inflammation: total white blood cell count [+0.5 × 10^3] and serum uric acid [+0.06 mg/dL] concentrations were higher, but did not reach the level of significance. Elevated uric acid levels among other things are associated with increase in blood pressure [32]. We demonstrated a significant increase in systemic inflammation concomitant with a significant increase in hs CRP [+0.4(0.09) mg/dL]. The serum levels of leptin were higher [+6.64 ng/mL; p =0.0008], a further indication of the potentiated systemic inflammation. Although post caloric total adiponectin [+0.98 μg/mL] and high-molecular-weight adiponectin [+598.43 μg/mL] were higher, they did not reach significance. This lack of significant increase despite a significant increase in fat mass would be indicative of adipose tissue dysfunction.

Caloric excess can also result in ectopic lipid deposition, especially in the liver and muscle, the consequence of which, among other things, is insulin resistance [33,34]. We found that our baseline intramyocellular lipid measurements compared to post caloric excess decreased, but intrahepatic lipid increased. These early reciprocal changes in the muscle and the liver of otherwise healthy subjects may be reflective of an adaptive increase in the oxidative capacity of the muscles (thus deceasing intramyocellular lipid) and the onset of hepatic insulin resistance [35,36]. There was also a significant increase in alanine transaminase concentration [+12.8(3.1) IU/L], an initial indicator for a functional impairment of hepatic function with non-alcoholic fatty liver disease [37]. We elucidated a significantly increased insulin resistance with elevation in fasting plasma glucose +7.0(0.6) mg/dL, fasting insulin +5.7(1.4) uIU/ml and HOMA-IR +1.6(0.5).

Along with the increase in body weight, body fat, waist circumference, visceral adipose tissue compartment, subcutaneous adipose cell size, systemic inflammation and insulin resistance, a loss of blood pressure control and endothelial dysfunction ensued in these subjects. This is in keeping with the abnormal circadian blood pressure variability and endothelial function being functional markers for an adverse cardiometabolic milieu [8,10]. In lean individuals with normal blood pressure, an increase in insulin levels abolished the nocturnal decrease in blood pressure and dampened the heart rate variability [38]. Increasing concentrations of serum insulin acutely enhance the gain of arterial baroreflex input to the muscle sympathetic nerve activity [39]. We elucidated a significant increase in 7-day mean systolic [+5.4(0.8)], diastolic [+1.7(0.1)] and pulse [+3.5(0.4) mm Hg] pressures, as well as an elevation of 7-day mean HR [+4.9(0.5) bpm].

Endothelium, a thin monolayer lining the inner surface of the blood vessels, is a receptor-effector organ which is responsive to both physical and chemical stimuli and maintains homeostasis not only by autocrine, but also paracrine and endocrine actions [40,41]. Endothelial dysfunction appears to be both an effect of increasing insulin resistance and cause in the pathogenesis of hypertension [42,43]. The activation of renin-angiotensin-aldosterone system [RAAS] is being recognized as being an underlying cause not only for hypertension, but also for insulin resistance and dyslipidemia [44]. Although we do not provide direct measurements, the demonstrated change in all three cardiometabolic parameters [blood pressure, glycemia and lipidemia] could be due to up regulation of adipose tissue RAAS. Subjects exhibited a decline in relative hyperemia index by 0.47(0.004) from initial 2.24(0.09) to 1.77(0.1), indicating endothelial dysfunction (impaired flow-mediated peripheral arterial tone).

This prospective study in free living healthy adults obtaining baseline and eight-week measurements validated our hypothesis that controlled caloric excess increases body fat mass, preferentially expands visceral adipose tissue, induces adipose cell dysfunction, potentiates systemic inflammation and worsens insulin resistance. These changes were also accompanied by persistent abnormal circadian blood pressure variability and endothelial dysfunction, both of which are functional correlates indicating enhanced cardiovascular risk [8,9]. Increased fat mass, elevated leptin concentration, stimulated hypothalamic sympathetic nervous system activity, which was functionally seen as abnormal circadian blood pressure variability and elevated heart rate. Expanded visceral adipose tissue with equivocal adiponectin change signified adipose tissue dysfunction, which potentiated systemic inflammation, exacerbated insulin resistance and induced resting endothelial dysfunction [9,12].

Interestingly, the three subjects who did not change their adipose cell size, did not also exhibit an adverse change in their glycemic indices. One of them did not develop any loss of BP control, while the other two did not exhibit endothelial dysfunction. In line with our overall hypothesis, the measures for circadian blood pressure variability correlated with systemic inflammation, which in turn was highly correlated with body weight and body mass index. This suggests that increased adiposity exacerbates systemic inflammation and (although not measured, plausibly due to increased elements of the renin-angiotensin-aldosterone system) induces loss of BP control. Fasting plasma glucose correlated with subcutaneous adipose tissue cell size suggesting that increased adipose cell size leads to insulin resistance. The correlation of serum triglycerides and alanine transaminase with visceral adipose tissue volume implicates dysglycemia and ectopic deposition of intrahepatic fat, respectively, with visceral adipose tissue accumulation. Additionally, the negative correlation of endothelial function with fasting plasma glucose, Total-C, LDL-C and triglycerides, explains endothelial dysfunction with dysglycemia and dyslipidemia.

#### Limitations

The absence of a free living control group followed over eight weeks without caloric excess is a limitation with this study. While weight gain accompanied with an alteration in body composition occurs naturally during various periods of life cycle [adolescence [45,46], freshman year at college [47,48], the period after marriage in men [49], pregnancy [50] and mid life in women [51,52], the change in weight and body composition in free living healthy adults over eight weeks is likely to be minimal. The average weight gain in young adults over time ranges from 0.2 to 0.8 kg per year [53-56]. In free living, healthy adult participants in this study, [mean age of 28 years; range 22-36 years], the change in weight over eight weeks for the control group would therefore be small compared to the significant mean weight gain of 7.4 kg [p = 0.001] with caloric excess. Since all of the assessments detailed are influenced to some degree by weight gain, it is also unlikely they would depict a significant change in the control group.

### Conclusions

The major finding from this study is that the seemingly benign changes resulting from a short period of controlled overeating were all appropriately, uniformly and significantly altered as hypothesized, equivocally elevating cardiovascular risk of the participants. While these changes in chronic disease states have been described piecemeal, this is a comprehensive demonstration of the early changes that occur with excess caloric consumption in healthy participants. Although in small numbers, this is a first prospective demonstration of the link between controlled caloric excess and a resulting increase in body fat, visceral adipose tissue mass, systemic inflammation, insulin resistance, loss of blood pressure control and endothelial dysfunction in humans.

### Competing interests

All authors have completed and submitted the ICMJE Form for Disclosure of Potential competing interest. No authors reported disclosures.

### Authors’ contributions

AKG was the medical investigator for the study, had access to the data and takes responsibility for the integrity of the data and the accuracy of data analysis. *Study concept and design*: AKG was the medical investigator for the EAT study, which was awarded to ER. AKG conceived the study, performed the analysis of the blood pressure and endothelial function data, initiated the manuscript and compiled the final version for submission. *Acquisition of data*: AKG. *Analysis and interpretation of data*: AKG, WDJ. *Drafting of the manuscript*: AKG. *Critical revision of the manuscript for important intellectual content*: AKG, DJ, ER, WDJ. *Statistical analysis*: WDJ, AKG. *Obtained funding*: ER. R01 DK060412 (EAT; PI ER) and P30DK072476 (NORC; PI ER. AKG, DJ and WDJ had no funding. No funding bodies had any role in study design, data collection and analysis, decision to publish, or preparation of the manuscript. *Administrative*, *technical*, *or material support*: Pennington Biomedical Research Center Cores. *Study supervision*: AKG. All authors read and approved the final manuscript.

### Additional contributions

The authors thank the participants without whom this study would not have been possible, the coordinators for this study who scheduled subjects through a very intensive study and the staff with the Pennington Biomedical Cores who helped obtain and compile the data.

### Funding/Support

The Fat Cell Size, Overfeeding and Ectopic Fat (EAT) study was funded R01 DK060412 (EAT; PI ER). The data collection was supported by Nutrition Obesity Research Center (NORC) P30DK072476 (NORC; PI ER).
